# Supplementary material for: Resolving the full spectrum of human genome variation using Linked-Reads
Source: Genome Res. 2019 Apr;29(4):635–45. doi: 10.1101/gr.234443.118 (PMC6442396; doi:10.1101/gr.234443.118)
Supplement: Supplemental Material [file supp_gr.234443.118_Supplemental_longranger-2.2.zip › longranger-2.2.tar.bz2!/lr2.2-public/lib/stan/cmdstan/stan/doc/api/html/iterator__typedefs_8hpp_source.html]

Stan: src/stan/gm/grammars/iterator\_typedefs.hpp Source File


|  |  |
| --- | --- |
|  | Stan  2.5.0  probability, sampling & optimization |


- Main Page
- Related Pages
- Namespaces
- Classes
- Files

- File List
- File Members

All Classes Namespaces Files Functions Variables Typedefs Enumerations Enumerator Friends Macros Pages

- src
- stan
- gm
- grammars

iterator\_typedefs.hpp

Go to the documentation of this file.

1 #ifndef STAN\_\_GM\_\_PARSER\_\_PARSER\_\_ITERATOR\_TYPEDEFS\_\_HPP

2 #define STAN\_\_GM\_\_PARSER\_\_PARSER\_\_ITERATOR\_TYPEDEFS\_\_HPP

3

4 #include <boost/spirit/include/version.hpp>

5 #include <boost/spirit/home/support/iterators/line\_pos\_iterator.hpp>

6

7 namespace stan {

8  namespace gm {

9  typedef std::string::const\_iterator input\_iterator\_t;

10  typedef boost::spirit::line\_pos\_iterator<input\_iterator\_t> pos\_iterator\_t;

11  }

12 }

13 #endif

stan::gm::pos\_iterator\_t

boost::spirit::line\_pos\_iterator< input\_iterator\_t > pos\_iterator\_t

**Definition:** iterator\_typedefs.hpp:10

stan::gm::input\_iterator\_t

std::string::const\_iterator input\_iterator\_t

**Definition:** iterator\_typedefs.hpp:9


---

[ Stan Home Page ]

*© 2011–2014,
Stan Development Team.*
